# Durable effect of pyrotinib plus capecitabine in HER-2+ breast cancer patient undergoing peritoneal dialysis: A case report and literature review

**DOI:** 10.3389/fonc.2022.1059670

**Published:** 2022-12-08

**Authors:** Xiangting Jin, Min Yan, Fanfan Li

**Affiliations:** Department of Oncology, The Second Affiliated Hospital of Anhui Medical University, Hefei, China

**Keywords:** breast cancer, end-stage renal disease, peritoneal dialysis, dose adjustment, pyrotinib

## Abstract

For patients with end-stage renal disease (ESRD), peritoneal dialysis (PD) and hemodialysis (HD) are important renal replacement treatments. Patients on dialysis usually have a high incidence rate of malignant tumors. In 2020, breast cancer has become the malignant tumor with the highest incidence rate in the world. Human epidermal growth factor receptor-2-positive (HER-2+) breast cancer accounts for 20%-30% of the total breast cancer patients. It is highly invasive and has a poor prognosis. Anti-HER-2 treatment is an important therapy for this type of cancer. There are few case reports of anti-HER2-targeted therapy in dialysis patients. We report a 56-year-old Chinese woman with breast cancer (cT3N1MX, Her-2+/HR-). She underwent peritoneal dialysis for 11 years since she had suffered end-stage renal disease. The clinician prescribed the regimen (pyrotinib 320mg qd + capecitabine 1g bid D1-D14 Q3W). The tumor was significantly reduced after 1 month of single administration of pyrotinib, and partially relieved after 2 months of pyrotinib + capecitabine. The main side effects were grade II hand foot syndrome and grade II diarrhea. This case shows that the combination of pyrotinib and capecitabine has potential therapeutic benefits in HER-2+ breast cancer patients with end-stage renal disease.

## Introduction

In recent years, the incidence rate of end-stage renal disease (ESRD) patients has increased year by year. Renal replacement therapy such as peritoneal dialysis (PD) and hemodialysis (HD) can significantly prolong the survival time of ESRD patients. With the extension of patients’ survival time, their risk of cancer will also increase (1). It is a great challenge for such patients who need long-term renal replacement therapy to receive anti-tumor drugs. According to the *Global Cancer Statistics 2020*, breast cancer has become the malignant tumor with the highest incidence rate in the world (2). Among them, human epidermal growth factor receptor 2 positive (HER-2+) breast cancer accounts for about 20%-30%, with strong invasion and poor prognosis (3). Anti-Her-2 treatment can significantly improve the survival of these patients. However, few cases of HER-2-positive breast cancer with long-term peritoneal dialysis treatment have been reported, and a few cases have been reported on hemodialysis (4). Due to the economy and convenience of peritoneal dialysis, the number of dialysis patients using PD in China far exceeds that in any other region in the world (5). PD patients accounted for about 14.4% of the total dialysis patients. For the treatment of ESRD tumor patients, in July 2017, the Italian Association of Medical Oncology (AIOM) and the Italian Society of Nephrology (SIN) jointly issued a proposal on the management of chemotherapy for patients with end-stage renal disease. It is necessary to adjust the drug dose according to the drug dialysis capacity and pharmacokinetics (6). In 2019, targeted treatment management recommendations for cancer patients with chronic kidney disease or hemodialysis were released. The anti-tumor treatment strategy of dialysis patients is basically the same as that of non-dialysis patients, and the renal clearance, dose and dialysability of chemotherapeutic drugs and targeted drugs need to be properly considered (7). This article reports a case of HER-2+ breast cancer who has been undergoing PD for a long time, and the successful individualized treatment of pyrotinib combined with capecitabine. To explore the treatment of breast cancer in ESRD patients and provide reference for clinicians.

## Case description

The patient is a 56-year-old female who has gone through menopause. In 2005, she was diagnosed with chronic nephritis, and in 2011, she was converted to chronic renal failure. At present, she has regular peritoneal dialysis once a day, and her blood creatinine is maintained at about 900umol/L. In May 2020, the patient underwent physical examination in the First Affiliated Hospital of Anhui Medical University and found right breast and right axillary mass. The diameter of the breast mass was about 3.0cm, the texture was hard, and the tenderness was (-). The doctor suggested hospitalization for further treatment. Because of the underlying disease, the patient refused further medical treatment. In April 2021, the right breast mass became significantly larger, about the size of a pigeon egg, with hard texture and tenderness (-). In July 2021, the patient underwent hollow needle biopsy of breast and axillary lymph nodes in the First Affiliated Hospital of Anhui Medical University. Right breast biopsy pathology: 3 gray and white cord like tissues, 0.6-1.6cm long and 0.1cm in diameter. Microscopic examination considered invasive breast cancer; Puncture pathology of right axillary lymph node: there were 3 gray and white cord like tissues, 1.0-1.5cm long, and abnormal cells were found in the fibrous lymph tissue by microscopic examination. Immunohistochemical results: ER (+, 1%), PR (+, 1%), HER-2 (3+), Ki-67 (+, 20%). Pathology: breast invasive carcinoma, non-specific type, who grade: Grade III, with ipsilateral axillary lymph node metastasis.

Then the patient was referred to our clinic (The Second Affiliated Hospital of Anhui Medical University) in order to receive further medical treatment.

**Figure 1:**
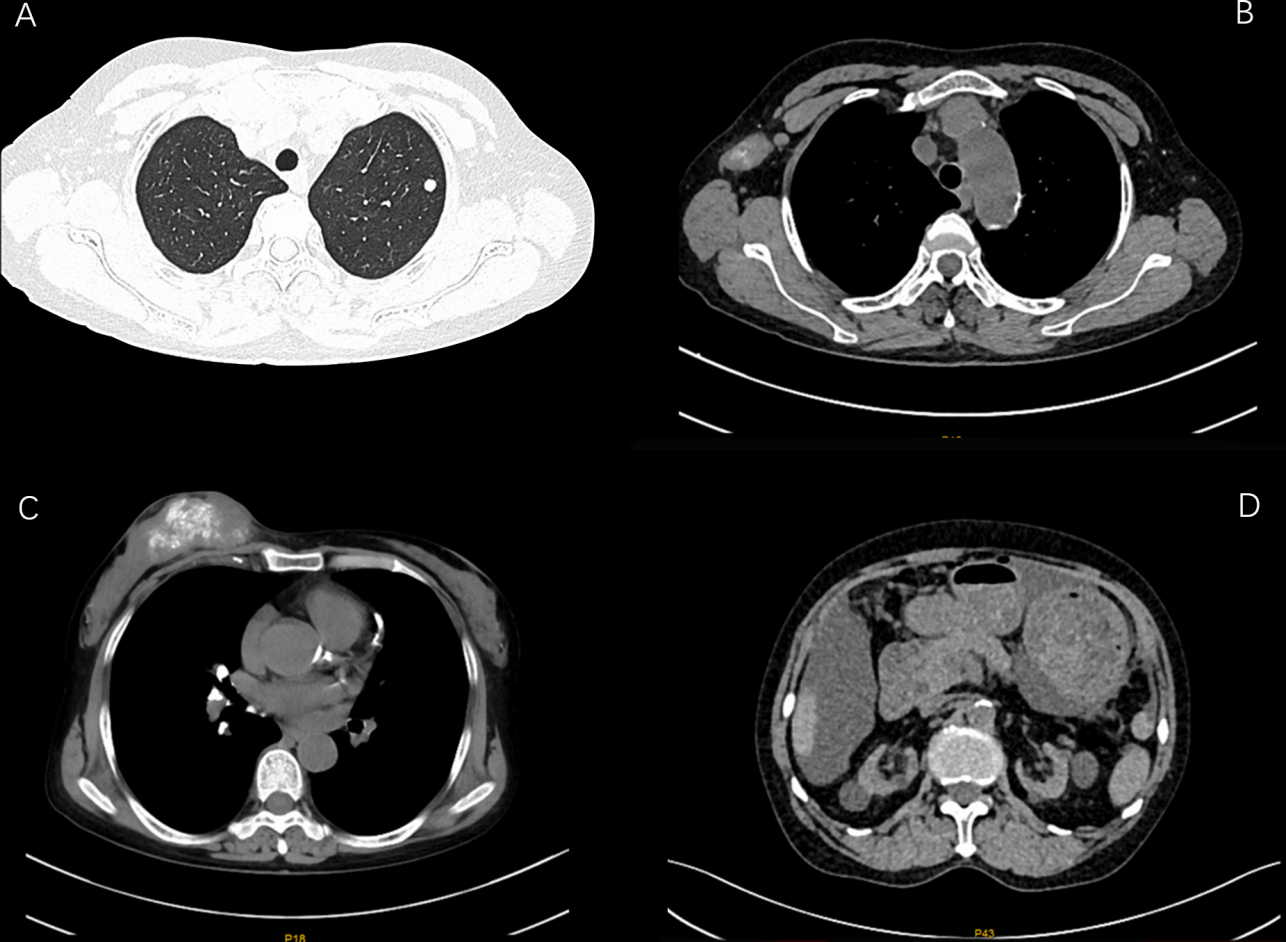
CT images of patient before anti -tumor treatment (**A**: lung nodules **B**: lymph nodes in the armpit **C**: massive soft tissue density with multiple calcifications in the right breast **D**: interval).

The laboratory examination (August 7, 2021) showed that hemoglobin was 68g/L, creatinine was 937umol/L, urea nitrogen was 20.78mmol/L, and the estimated glomerular filtration rate was 4.93ml/min.1.73m^2^. The results of computed tomography (CT) plain scan of chest and abdomen (**Figure 1**): Massive soft tissue density with multiple calcifications in the right breast. There were several small and medium lymph nodes in the right axilla. Several nodules were seen in both lungs. Multiple small and medium lymph nodes with local calcification appeared in the mediastinum and in the bilateral hilum.

## Clinical diagnosis

Right breast invasive carcinoma (HER-2+, HR-,cT3N1MX); pulmonary nodules(high likelihood of secondary lung malignancy); chronic kidney disease stage V; Peritoneal dialysis; renal anemia.

## Treatment process

**Figure 2:**
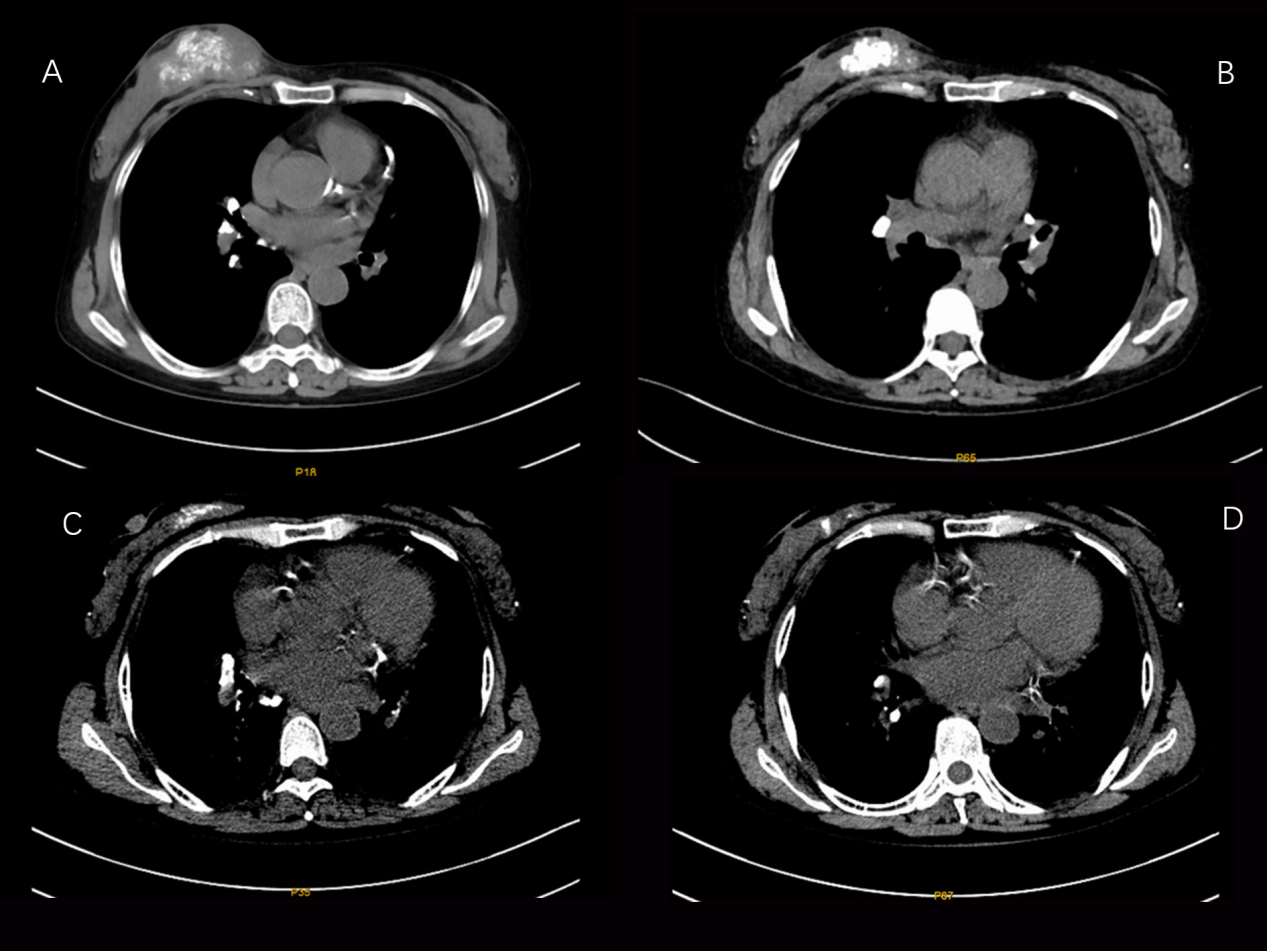
CT images of changes in breast tumor lesions in patients during the treatment period from August 6, 2021 to February 25, 2022 (**A**:2021-08-06 **B**:2021-09-25 **C**:2021-12-17 **D**: 2021-02-25).

**Figure 3:**
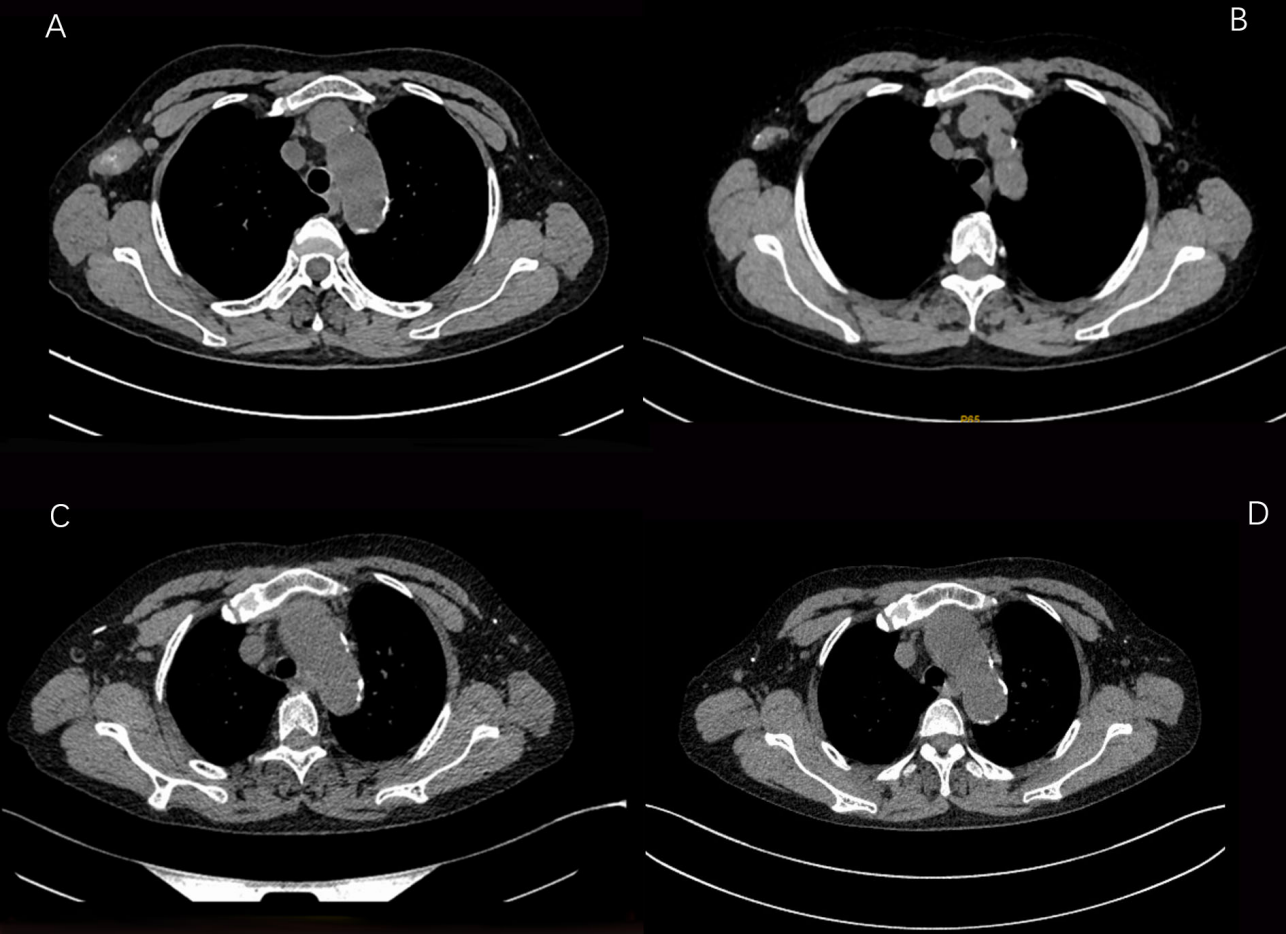
CT images of changes in the axillary lymph node lesions of the patient during the treatment period from August 6, 2021 to February 25, 2022 (**A**:2021-08-06 **B**:2021-09-25 **C**:2021-12-17 **D**: 2021-02-25).

According to the imaging and immunohistochemical results of the patient’s initial diagnosis, we believe that the patient is more likely to have stage IV breast cancer. According to the patient’s own recollection, no pulmonary nodules were found before the patient developed breast cancer. Although the diameter of the pulmonary nodule is less than 10mm, We believe that the lung nodules are likely to be metastatic tumors originating from the breast. At the same time, multidisciplinary expert consultation recommended no surgical treatment considering that the patient needed to undergo peritoneal dialysis once a day and the patient’s poorer hematological parameters. On August 11, 2021, the patient began to take pyrotinib 320mg qd orally. Three days later, the patient experienced an increase in the number of stools (4-5 times a day), loose stools, and no purulent bloody stools (according to the common Terminology criteria for adverse events of the National Cancer Institute of the United States, CTCAE 5.0.It was evaluated as grade II). It relieved After oral administration of montmorillonite. One week after the patient’s oral administration of pyrotinib, the palpation of the right breast mass and the right axillary mass were significantly reduced and softened. On September 25, 2021, the CT scan of the chest, abdomen, and pelvis showed that the right breast mass and the right axillary lymph node were significantly reduced (**Figures 2**, **3**). Calcium and other indicators remained basically stable (**Table 1**). On September 29, 2021, oral capecitabine Capecitabine 1g bid D1-14 Q3W was added. On November 1, 2021, the patient developed desquamation, redness and swelling of the extremities, and pain to the touch, accompanied by mild nausea and decreased appetite (CTCAE grade II). External application of urea and vitamin E ointment has improved the numbness and pain at the ends of hands and feet, and the tolerance is acceptable. As of the last review on July 3, 2022, the patient’s efficacy was evaluated as partial response according to the response evaluation criteria in solid tumors (RECIST) version 1.1, and the progression-free survival was 11 months.

**Table 1:** Detection indicators of peripheral blood of patients.

DATE	BUN (mmol/L)	CREA(μmol/L)	eGFR(ml/min.1.73m^2^)	UA(μmol/L)	RBC(×10^12^)	Hb(g/L)	ALB (g/L)	Ca(mmol/L)	IP (mmol/L)
2021/8/7	20.78	937	4.93	367	2.23	68	28.6	2.64	1.62
2021/8/16	17.3	925	5.00	301	2.42	74	29.6	2.58	1.73
2021/9/25	15.71	872	5.36	359	2.29	73	26.4	2.58	1.52
2021/10/30	15.37	766	6.22	402	2.08	67	26.9	2.59	1.34
2021/12/18	19.49	834	5.62	412	1.92	63	27.2	2.6	2.04
2022/1/23	16.28	818	5.75	316	2.02	65	29.0	2.63	1.38
2022/2/26	16.34	833	5.63	332	2.15	69	30.4	2.66	1.55
2022/5/11	26.36	804	5.86	410	2.12	69	31.1	2.33	1.23
2022/7/11	14.68	732	6.53	326	3.75	124	26.9	2.18	1.51

## Discussion

As the incidence of ESRD grows in the world, more patients are receiving dialysis (8). Multiple studies have shown that ESRD patients have an increased risk of developing cancer with or without dialysis (9–13). Possible mechanisms include: altered DNA repair, impaired immune system function, reduced antioxidant defenses, chronic infection and inflammation, and accumulation of carcinogenic compounds (14). Lee,M et al. think that (15), There was no significant difference in cancer incidence among patients receiving renal replacement therapy such as peritoneal dialysis, hemodialysis, and kidney transplantation. The U.S. Renal Data System shows an increased risk of cancer, including breast cancer, in hemodialysis (HD) patients. Between 1996-2009, 3552 women with HD were diagnosed with breast cancer, 42% higher than the general population (16). The treatment of breast cancer with antitumor drugs will bring a new challenge to these patients who have been treated with renal replacement therapy for a long time. Issues such as how to adjust doses based on pharmacokinetic (PK) and pharmacodynamic (PD) parameters, as well as the timing of dosing associated with dialysis treatment, all affect the optimal anticancer efficacy of such patients.

Regarding the treatment of HER-2-positive breast cancer, the CLEOPATRA study suggests the use of trastuzumab + pertuzumab + docetaxel for previously untreated HER-2-positive locally advanced or metastatic breast cancer Compared with the trastuzumab + docetaxel regimen alone, its PFS (18.7 months vs 12.4 months), median OS (57.1 months vs 40.8 months) were significantly increased, and the 8-year overall survival rate was 37%, 8-year overall survival rate of 37% (17). Subsequently, the Chinese bridging study PUFFIN once again confirmed the effectiveness of the dual-target combination chemotherapy regimen, with an ORR of 79% and a 31% reduction in the risk of disease progression (18).Therefore, the National Comprehensive Cancer Network (NCCN), European Society of Medical Oncology (ESMO), American Society of Clinical Oncology (ASCO) and Chinese Society of Clinical Oncology (CSCO) guidelines recommend pertuzumab combined with trastuzumab and chemotherapy as Standard regimen for first-line treatment of HER-2-positive breast cancer. Antibody-conjugated drugs such as T-DM1 and T-DXd are the second-line treatment options for foreign HER-2-positive breast cancer, and lapatinib has also shown good efficacy in second-line and subsequent treatments. However, due to the poor economy and accessibility of the above drugs in Chinese patients, they have not been widely used in China. Pyrotinib is a self-developed multi-target tyrosine kinase inhibitor in my country. The PHOEBE study showed that in HER-2-positive advanced breast cancer patients who failed trastuzumab treatment, the pyrotinib + capecitabine group was more Lapatinib + capecitabine was significantly more effective: ORR (67.2% vs 51.5%), median PFS (12.5 vs 6.8 months), and reduced risk of disease progression61% (19).

However, regardless of whether it is a chemotherapeutic drug or an anti-HER-2 targeted drug, its various clinical studies have not conducted pharmacokinetic studies in ESRD dialysis patients, and the existing data cannot provide effective dosage guidance for ESRD patients. BEDNAREK et al. suggested that renal clearance of drugs and their metabolites in ESRD patients during HD and PD depends on their diffusivity through semipermeable membranes and is limited by their protein binding capacity. Drugs with low molecular weight and poor protein binding are easily removed by the dialysis membrane, so drugs such as cyclophosphamide, fluorouracil, and capecitabine should not be used during the patient’s dialysis time (20). At present, the application of many chemotherapeutic drugs in ESRD is mostly a small number of case reports, and most of them are used in HD, and there are very few reports in PD. In July 2017, the Italian Association of Medical Oncology (AIOM) and the Italian Society of Nephrology (SIN) jointly issued a consensus on the management of chemotherapy in patients with end-stage renal disease: capecitabine has a certain safety in a small number of reports, but Contraindication is recommended in patients with GFR <30ml/min to avoid increased incidence of grade 3-4 adverse events. For anthracyclines, the majority of doxorubicin users are patients with hematological diseases who receive HD, and standard doses are safe and effective. Epirubicin was only used in one case, and it was reported to be safe and effective. For cyclophosphamide, a 30% reduction is recommended. For paclitaxel, some small studies showed no significant difference compared with normal renal function, so no dose adjustment was required. Docetaxel is recommended to reduce the dose to 65mg/m^2^ in combined use.

In 2019, the Italian Association of Medical Oncology (AIOM) and the Italian Society of Nephrology (SIN) jointly issued a consensus on the management of cancer targeted therapy: Trastuzumab and Pertuzumab are recombinant humanized monoclonal antibodies whose metabolism Elimination of immune globulin clearance by the reticuloendothelial system is not recommended for dose reduction in patients with mild to moderate chronic kidney disease, and data are insufficient in ERSD and dialysis patients. TKIs such as neratinib, pyrotinib are strongly bound to proteins and eliminated by the liver and excreted mainly in the feces (metabolites) (21, 22). Therefore, HD and PD are not expected to enhance the clearance of TKIs. At the same time, it has been reported that a HD breast cancer patient received lapatini for more than 3 years, and there was no obvious increase in toxicity.

For this patient, combined with the patient’s wishes, we believe that compared with macromolecular targeted drugs such as trastuzumab and pertuzumab, pyrotinib has irreversible binding to the action site, stable action, and as an oral drug, it has the advantages of good tolerance and easy penetration through the blood-brain barrier (23).Compared with similar TKI drugs such as neratinib and lapatinib, it is more accessible and economical for Chinese patients. Considering his renal function, pharmacokinetics, pharmacodynamics and safety of various drugs, we chose pyrotinib 320mg qd + capecitabine 1g bid D1-D14 Q3W regimen for anti-tumor therapy for this patient. Adherence was better in patients with a dual oral drug regimen.

The patient received single-agent pyrotinib in the first month, and its side effects were mainly diarrhea, which improved after oral administration of montmorillonite powder. The patient started adding capecitabine from the second month, and the extremities were desquamated, red and swollen, and painful to the touch, accompanied by mild nausea and decreased appetite, and then the capecitabine dose was reduced to 0.5 g in the morning and 0.5 g in the evening D1 -14 Q3W, and external use of urea vitamin E cream, the numbness and pain at the ends of hands and feet improved. No neutropenia or thrombocytopenia found. The patient continued on peritoneal dialysis as planned. During the follow-up chemotherapy and follow-up period, the serum creatinine and urea values of the patients had no significant changes compared with those before chemotherapy, indicating that the pyrotinib + capecitabine regimen had no significant effect on the renal function of the patients. On 2021.08.06, 2021.09.25, 2021.12.17, 2022.02.25, 2021.05.10, the complete CT examination showed that the tumor lesions of the patients were significantly reduced during the course of treatment, and the patients were generally in good condition during the follow-up period. To date, no disease progression has been detected.

The pyrotinib + capecitabine regimen did not show any significant adverse effects on renal function in peritoneal dialysis patients with ESRD; The patient needed dose reduction treatment due to capecitabine toxicity leading to hand-foot syndrome. After treatment, the patient’s tolerance was acceptable. During the course of treatment, the patient’s lesions were significantly reduced, and the effect reached PR. We believe that the pyrotinib + capecitabine regimen may be a good treatment option as a first-line treatment for advanced HER-2-positive breast cancer. ESRD is not a contraindication for systemic treatment of breast cancer. Such patients can achieve corresponding individualized treatment through renal function monitoring and regular dialysis.

## Data availability statement

The datasets presented in this study can be found in online repositories. The names of the repository/repositories and accession number(s) can be found in the article/supplementary material.

## Ethics statement

The study involving human participant was reviewed and approved by ethics committee of the Second Affiliated Hospital of Anhui Medical University. The patient provided her written informed consent to participate in this study. Written informed consent was obtained from the individual for the publication of any potentially identifiable images or data included in this article.

## Author contributions

LF conceived and designed the study. JX and YM collected the data and wrote the manuscript. All authors contributed to the article and approved the submitted version.

## Funding

This study was funded by the Clinical Research Incubation Program of the Second Affiliated Hospital of Anhui Medical University (2020LCYB13).

## Conflict of interest

The authors declare that the research was conducted in the absence of any commercial or financial relationships that could be construed as a potential conflict of interest.

## Publisher’s note

All claims expressed in this article are solely those of the authors and do not necessarily represent those of their affiliated organizations, or those of the publisher, the editors and the reviewers. Any product that may be evaluated in this article, or claim that may be made by its manufacturer, is not guaranteed or endorsed by the publisher.
